# Veratrum Viride

**Published:** 1853-08

**Authors:** John S. Wilson

**Affiliations:** Airmount, Clarke county, Alabama


*A brief Summary of my Experience with the Veratrum Viride.* By John S. Wilson, M. D., of Airmount, Clarke county, Alabama.—It is not my design to give a minute and tedious detail of cases ; I will only mention some of the diseases in which I have used the medicine under consideration—the mode of administration, and the result, together with such general remarks as may be suggested to me in common with each case.

At the beginning of the present year, I succeeded in obtaining some of the root of the veratrum viride, after several ineffectual efforts : from this I made a tincture, according to the directions of Dr. Norwood, by adding ½ lb. of the root to 16 oz. of undiluted alcohol. This tincture I have used in the following diseases—viz : Pneumonia, pleuro-pneumonia, bronchitis, broncho-pneumonia, pericarditis, cerebro-spinal meningitis, epileptiform convulsions, acute rheumatism, general dropsy ; and in fevers —typhoid, remittent and puerperal. Of the thoracic diseases, sixteen were pulmonary and one cardiac : of meningitis, convulsions, acute rheumatism, and dropsy, there was one case each : of the fevers, three were typhoid, one puerpal, and several remittent. Of the sixteen pulmonary cases, all recovered save one, and this was complicated by the supervention of acute rheumatism. The case of pericarditis had resulted in effusion, before it was put under treatment, and terminated fatally : still the veratrum exerted its wonted control over the pulse, reducing its frequency from 140 to 60. The case of cerebro-spinal meningitis was also fatal ; but as in the other case, the remedy exerted a marked control over the pulse, and for a while there was a flattering amelioration of all the symptoms.

The subject of the convulsions was an anemic boy of 10 or 12 years of age; the spasms were very frequent and extremely severe, every paroxysm apparently putting the life of the patient in imminent danger: the veratrum, to reduce the frequency of the pulse, and ether inhalations, to quiet the spasms, were the remedies almost exclusively relied upon; and these indications were fulfilled in the happiest possible manner, snatching the little sufferer from the very jaws of death.

The case of rheumatism occurred in a little boy about 5 years of age; it attacked the knee joint, was very severe, and was treated principally with colchicum and veratrum; but the former medicine did not succeed well until the latter was added to prescription. Under this treatment the disease was subdued in less than six days, instead of running "six weeks," according to the dictum of Dr. Warren.

The case of dropsy was fatal, the patient being moribund when the veratrum was commenced.

We now come to that interesting class of diseases—fevers. Of the three typhoid cases, one terminated favorably in twelve days; but the early convalescence in this case was not attributed to the veratrum, as this was not made a principal remedy, and as the fever was unusually mild. The other two cases were severe and protracted, one continuing three and the other six weeks. Having much confidence in the veratrum, it was relied upon almost exclusively in the treatment of these two cases, the prescriptions being complicated as little as possible. In the case that terminated in convalescence in three weeks, slight ptyalism supervened, from the use of small doses of calomel in conjunction with the veratrum.

The general plan of treatment which I have adopted in typhoid fever is to give quinine freely for the first few days, or until the typhoid symptoms are fully developed; I then resort to small doses of calomel and the veratrum, experience having taught me that nothing can be gained by pushing the quinine, *after the disease has become distinctly typhoid.* In giving the veratrum, I generally prescribe 4 gtt. every three hours, for an adult, to be increased 1 gtt. each dose, until a decided effect is produced, either upon the pulse or stomach. I have never been able to carry the dose higher than 14 gtt., and seldom higher than 8 gtt. ; and I have even known 2 gtt. to produce a very manifest effect on the pulse of a little girl three years of age. I have never known it to fail in reducing the frequency of the pulse when it produced nausea, but I have frequently succeeded in reducing the pulse, without any nausea, by beginning with very small doses, and increasing gradually.

Having mentioned the disease in which I have used the veratrum, and the mode of administration, I conclude with a few general remarks

It will be seen by reference to the figures given, that my experience with it has been more extended in pulmonary diseases than in any other class—sufficiently so, I think to speak with some degree of confidence. As already stated, only one of those cases proved fatal, and that was complicated. Some of the cases were severe and alarming, as much so as any I have ever seen ; and one of the cases of pneumonia was accompanied with active pulmonary hemorrhage. With these facts before me, I can but express a very favorable opinion of the veratrum in pneumonia and bronchitis. Before the introduction of this remedy, I treated the diseases mentioned with antimonials, and generally with success ; but every physician of experience knows that pneumonia is not unfrequently complicated with a degree of intestinal irritation which forbids the use of tart. emetic on the Rasorian plan, or, indeed, in any way capable of subduing the pulmonary inflammation : and even where this intestinal complication is not an essential feature of the disease, it is well known that the free use of tart antimony will produce it, together with other grave, and even fatal symptoms. The veratrum is not obnoxious to those objections ; and having in addition to the important negative advantage, the positive one of being more prompt and certain in its effects, than the antimony, I think it entitled to the preference ; and I think it possible that time and experience will confirm its claims, and that it will be recognized as the great desideratum in the treatment of pulmonary diseases. In this class of diseases I have found it to act promptly, not only as a sedative, but also as an expectorant and anodyne ; and I would here remark, that I consider its primary acton, that which gives it its great value, sedative —while its secondary effects are expectorant, emetic, sudorific, and anodyne.

As to its use in fever, I have but few more words, as it is my design to confine myself mostly to facts, and as my experience with it in this class of diseases has not been extensive. As before intimated, the veratrum was made the principal remedy in the treatment of two of the typhoid cases—no other remedies being used except blisters to the abdomen, cold sponging, spts. mindereri, and in one case small doses of calomel. If I may venture an opinion as to the virtues of veratrum in typhoid fever, from its effects in those two cases, it is this: Its powers in abbreviating the fever are doubtful; but its control over the pulse, the reduction of excessive arterial action, and the consequent mitigation of all the more distressing symptoms, are, comparatively, certain—far more so than any remedy previously in use. But, while I express myself thus favorably, I do not think the veratrum is applicable to *all* cases of typhoid fever; for I would be averse to its use in cases attended with extreme debility and great prostration of the vital powers: If I were to resort to it at all in cases of this kind, it would be in doses too small to produce nausea and prostration, observation having taught me that the patient must have a considerable degree of recuperative energy to withstand its depressing effects, unless it is used with great caution.—*South. Med. and Surg. Jour.*

